# Exploring the role of hand gestures in learning novel phoneme contrasts and vocabulary in a second language

**DOI:** 10.3389/fpsyg.2014.00673

**Published:** 2014-07-01

**Authors:** Spencer D. Kelly, Yukari Hirata, Michael Manansala, Jessica Huang

**Affiliations:** ^1^Neuroscience Program, Department of Psychology, Colgate UniversityHamilton, NY, USA; ^2^Center for Language and Brain, Colgate UniversityHamilton, NY, USA; ^3^Department of East Asian Languages and Literatures, Colgate UniversityHamilton, NY, USA

**Keywords:** multimodal, gesture, speech, L2, phoneme, vowel length contrast

## Abstract

Co-speech hand gestures are a type of multimodal input that has received relatively little attention in the context of second language learning. The present study explored the role that observing and producing different types of gestures plays in learning novel speech sounds and word meanings in an L2. Naïve English-speakers were taught two components of Japanese—novel phonemic vowel length contrasts and vocabulary items comprised of those contrasts—in one of four different gesture conditions: Syllable Observe, Syllable Produce, Mora Observe, and Mora Produce. Half of the gestures conveyed intuitive information about syllable structure, and the other half, unintuitive information about Japanese mora structure. Within each Syllable and Mora condition, half of the participants only observed the gestures that accompanied speech during training, and the other half also produced the gestures that they observed along with the speech. The main finding was that participants across all four conditions had similar outcomes in two different types of auditory identification tasks and a vocabulary test. The results suggest that hand gestures may not be well suited for learning novel phonetic distinctions at the syllable level within a word, and thus, gesture-speech integration may break down at the lowest levels of language processing and learning.

## Introduction

The present study explored the question of whether vocabulary and auditory learning in a second language (L2) can be aided by different types of multimodal training, particularly, involving observing or imitating different types of bodily actions. The study is guided by the general view that language processing and learning is fundamentally a *whole body* experience. Indeed, as the current special issue highlights, speech is inherently and systematically embedded within a variety of multimodal behaviors—visual, tactile, and proprioceptive—that are not merely peripheral parts of language, but together with speech, holistically *constitute* language (Clark, 1996; Calvert et al., 2004; McNeill, 2005). There is a rich tradition of research exploring this question in the context of how mouth and lip movements contribute to language processing and learning (sparked by the classic work on the McGurk effect), but more recently, researchers have begun to consider the role that other parts of the body play as well. The present study focuses on one such prominent behavior: hand gesture.

Co-speech gestures are spontaneous hand movements that naturally and ubiquitously accompany speech across different ages, languages, and cultures. Researchers have theorized that gesture and speech stem from the same conceptual starting point in language production, and thus form a fundamentally integrated system of communication (McNeill, 1992; Kendon, 2004). In addition to their role in producing language, co-speech gestures play an active role for language comprehension at various linguistic levels, such as pragmatic, semantic, and syntactic levels (Kelly et al., 2010; Hostetter, 2011; Holle et al., 2012), and this integrated relationship manifests in learning and memory as well (Thompson, 1995; Kelly et al., 1999; Feyereisen, 2006; Straube et al., 2009). Moreover, the role of hand gestures is not limited to one's native language, but they assist in adults' L2 learning as well (Quinn-Allen, 1995; Sueyoshi and Hardison, 2005; Kelly et al., 2009). Kelly et al. (2009), for example, examined the role of iconic gestures in L2 vocabulary learning, and found that English-speakers learned Japanese words better when iconic gestures, such as a drinking gesture, accompanied spoken Japanese words, e.g., *nomu* “drink,” compared to when those words were presented alone.

Although most of the research on the integration of gesture and speech focuses on higher levels of analysis (e.g., semantic and pragmatic), there is evidence that the two modalities may also be integrated at lower phonological levels as well (Gentilucci, 2003; Bernardis and Gentilucci, 2006; Krahmer and Swerts, 2007; Hubbard et al., 2008; Biau and Soto-Faraco, 2013). For example, Krahmer and Swerts (2007) showed that, when people produced particular words with beat gestures (which convey rhythmic and prosodic information) in a sentence, they produced those specific words with increased duration and increased pitch height. In addition, when the same spoken words were dubbed into video stimuli with or without those beat gestures, listeners-viewers perceived those words to be more acoustically prominent when presented with the gestures than without them. Moreover, even within the processing of a single word, gestures affect acoustic features of speech. For example, Gentilucci (2003) showed that viewing different sized gestures made toward different sized objects modulated lip aperture and voice peak amplitude of a speaker producing individual syllables of “BA” and “GA.” In this way, gestures can have a significant impact on speech production and comprehension—both in a sentence and word context—even at pre-semantic stages of processing.

**Figure 1:**
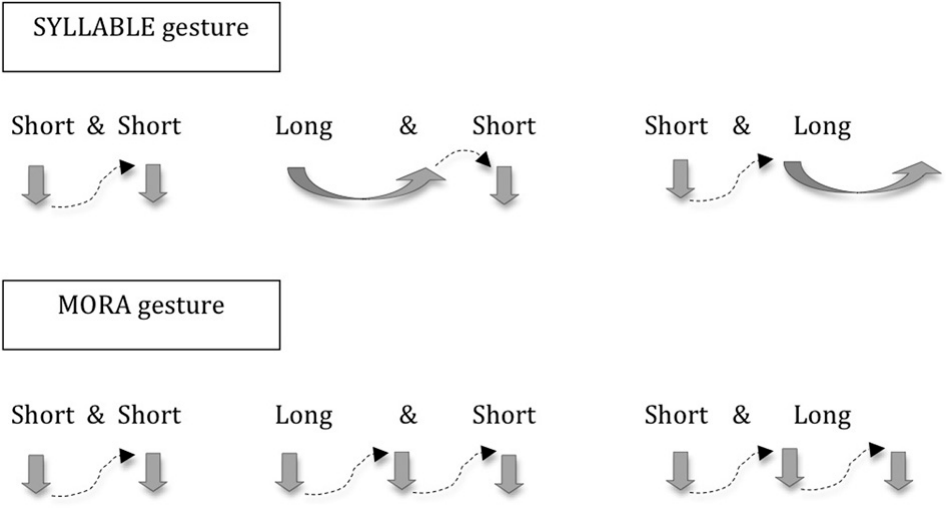
**Two types of hand gestures used in the present experiment**.

Returning to the domain of L2 learning, this opens up an interesting new line of inquiry. Given that other types of visual input (e.g., lip and mouth movements) are well known to help with novel L2 speech perception and learning (Hardison, 2003, 2005; Wang et al., 2008; Hirata and Kelly, 2010), it makes sense to ask what role that hand gestures play in this process as well. In one of the only studies on the topic, Hirata and Kelly (2010) examined the role of co-speech gestures in auditory learning of Japanese vowel length contrasts. Vowel length is phonemic in Japanese, e.g., [kedo] “but” with a short vowel [e] vs. [ke:do] “slight degree” with a long vowel [e:], and L2 learners have difficulty distinguishing these vowel length contrasts (Hirata et al., 2007; Tajima et al., 2008). In Hirata and Kelly (2010), English-speaking participants saw videos of Japanese speakers producing Japanese short and long vowels with and without hand gestures that represented the rhythm of those vowels, i.e., the Syllable gesture in Figure 1. A short vertical chopping movement was used for a short vowel, and a long horizontal sweeping movement was used for a long vowel^1^. Contrary to their predictions, participants in the speech-gesture condition did not learn to perceive the short/long vowel contrasts any better than those in the speech alone condition. The authors interpreted this result as hand gestures not playing a role at the segmental phonology level, suggesting a lower limit of speech-gesture integration. However, as the authors also pointed out, it is possible that there might be more effective types of gestures and methods of training. Therefore, the present study explored effects of another type of gesture, i.e., the Mora gesture in Figure 1.

The mora is a fundamental unit of timing for Japanese, and a series of moras create temporal beats of roughly equal intervals. The mora is like the syllable but is duration sensitive: long vowels are represented in two moras, or two equal rhythmic beats (Ladefoged, 1975; Port et al., 1987; Vance, 1987; Han, 1994). This mora rhythm is counter-intuitive for English speakers because a long vowel is still one syllable, or one beat. However, the Mora gestures that visually represent two short beats, rather than one extended one, for a long vowel might actually help native-English speakers learn this new mora-based rhythmic system, and ultimately help them hear the short and long vowel distinction better. Indeed, given research showing that “mismatching gestures” complement speech to facilitate learning (Goldin-Meadow, 2005), these counter-intuitive Mora gestures, which hint at a different rhythmic concept, may promote learning by highlighting new and useful strategies.

Another consideration for the design of the present study was that, while Hirata and Kelly (2010) asked learners to observe hand gestures, there is a good reason to believe that observing and imitating, i.e., *producing*, gestures oneself may have a more direct impact on learning than just observing them. There is tradition of research, now labeled “embodied cognition,” showing that physically producing actions leads to better learning and memory than just observing them alone (Saltz and Donnenwerth-Nolan, 1981; Cohen, 1989), and recent research has demonstrated that producing gestures is better than just observing them in various instructional settings (Goldin-Meadow et al., 2009; Goldin-Meadow, 2014). In the context of learning an L2, researchers have shown that gesture and speech interact during L2 speech production (for reviews, see Gullberg, 2006; Gullberg et al., 2008) and producing gestures plays a facilitative role in the learning process (Asher's, 1969, “Total Physical Response” technique). A more recent study showed that imitating iconic co-speech gestures helps adults to remember the meaning of words in an invented language more than imitating unrelated hand movements (Macedonia et al., 2011).

With specific regard to Japanese vowel length contrasts, Roberge et al. (1996) taught learners of Japanese to produce hand gestures to differentiate Japanese short and long vowels and observed that these gestures helped learners make significant progress in their short and long vowel production. The explanation of this finding was that by extending the muscles of the arm, the motor system of the arm “resonated” with the vocal motor system, and this made it easier to produce the novels sounds after training. Indeed, research in neuroscience has revealed direct and facilitative connections between producing one's own speech sounds and manual gestures, and this link is also systematically related to the comprehension of the same sounds and gestures produced by others (Bernardis and Gentilucci, 2006; Montgomery et al., 2007; Willems and Hagoort, 2007). These findings suggest that although Hirata and Kelly (2010) showed no unique effect of observing gestures on L2 learners' auditory abilities, *producing* them, in contrast, may be more effective in enabling learners to distinguish the vowel length contrasts.

What role do these new methods of training with co-speech hand gestures play in the process of learning to hear difficult L2 sound distinctions and mapping them onto the meaning of new words? Although L2 researchers have traditionally studied phoneme and semantic learning separately, it is important to note that there are close ties between these two abilities when learning an L2. For example, Bundgaard-Nielsen et al. (2011) examined the relationship between the ability to perceive Australian English vowels and vocabulary size by Japanese learners of English, and found that the more accurate their perception, the larger the size of vocabulary. Wong and Perrachione (2007) examined the auditory-vocabulary relationship with learning of Mandarin pseudo words by native English speakers, and found that the learners' ability to attach meaning to the sounds of Mandarin tones (i.e., high-level, rising, and falling) depended on their *initial* auditory ability to identify non-lexical pitch patterns. For Japanese vowel length contrasts, a preliminary finding suggested that a group of learners' very early auditory identification of these contrasts—even before learning meaning of words—enabled greater vocabulary learning as compared with those who learned word meanings first and then were trained to hear these contrasts later (Hirata, 2007).

The ability to hear isolated syllables or words as tested in the above studies, however, might not be a complete measure of learners' ability to perceive fluent sentences. In order to accurately perceive short and long vowels of Japanese, for example, learners must be able to normalize speaking rate of utterances and compare duration of a target vowel with other vowels in a sentence because the duration of “short” or “long” is a relational concept (Hirata, 2004a; Hirata et al., 2007). This *generalized auditory ability* may not necessarily develop if learners are trained only on words in isolation (Hirata, 2004b). Thus, the extent to which various auditory abilities relate to attaching meaning to novel L2 words in a sentence context is still unclear in extant literature.

Given this background, the present study examined effects of multimodal L2 training on auditory abilities through two tasks: the first was *an auditory identification test* in which participants were asked to identify the words they had learned in training (e.g., [seki] “seat” with two short vowels and [seːki] “century” with one long and one short vowel) vs. untrained words that were similar in syllable compositions but differed in the length of vowels (e.g., [sekiː] (nonsense word) with one short and one long vowel). The second task was *an auditory generalization test* in which participants were asked to identify the length of vowels in novel words that they did not hear during training in sentences of different speaking rates. In addition to these two auditory tests, we conducted a vocabulary test consisting of the trained words, e.g., [seki]/[seːki], and the novel words that differ in vowel length, e.g., [sekiː]. This vocabulary test measured how well learners remembered the translation of the trained words, as well as their ability to detect distractor words that differed in length of one of the vowels. The present study examined these three measures in four groups that each went through a different type of multimodal training, (1) Syllable-Observe, in which participants observed the Syllable gesture, (2) Syllable-Produce, in which they observed and produced the Syllable gesture, (3) Mora-Observe, in which they observed the Mora gesture, and (4) Mora-Produce, in which they observed and produced the Mora gesture. The study explored the extent to which these different training types yielded differential results in the above three measures.

In summary, the present study examined the following questions:

1. Given Goldin-Meadow's (2005) work on gesture-speech “mismatches,” does the mora gesture yield a greater auditory and vocabulary learning than the syllable gesture?
2. Given the literature in embodied learning (Saltz and Donnenwerth-Nolan, 1981; Cohen, 1989), does imitating gestures yield a greater amount of L2 auditory and vocabulary learning than just observing them?
3. How do effects of the different types of multimodal L2 training manifest in the ability to learn meaning of new words in relation to various auditory abilities, such as differentiating trained and untrained words in a memory task, and identifying short and long vowels in novel words in sentences spoken at different speaking rates?

There are several scenarios as to how our multimodal training may manifest among our different learning measures. For example, if producing gestures contributes to more robust and generalized auditory learning than observing gestures, we predict that participants with the former training would show significantly higher scores on all of these tests. Alternatively, the former group might show significantly higher auditory sentence test scores than the latter group, while both groups score the same on the vocabulary test. There are other possible outcomes as well, but because these were exploratory analyses, we did not have specific a priori predictions about how the four groups' performance would differ on the different dependent measures. However, administering these multiple tests may help tease apart the precise effects of the multimodal input they had received.

## Methods

### Participants

Eighty-eight undergraduate students at a liberal arts college in the Northeastern U.S. participated in the study. They were monolingual native speakers of English (males and females) with no knowledge of Japanese language, with an age rage of 18–23. None of these participants had extensive auditory input of Japanese or grew up in bilingual family environments. Participants' formal study of foreign languages included less than 6 years of French, Spanish, German, Italian, Russian, Mandarin Chinese, Arabic, Hebrew, Latin, and Greek. Any participant who had more than 6 years of continuous music training (as screened by a questionnaire) was not included because such musical training is known to affect auditory learning of foreign languages (Sadakata and Sekiyama, 2011). Participants were also screened to be right-handed because the training involved using the right hand in imitating the hand gesture on the computer screen.

Participants were randomly assigned to one of the four training conditions: Syllable-Observe (SO), Syllable-Produce (SP), Mora-Observe (MO), and Mora-Produce (MP) (*n* = 22 in each condition).

### Overall structure of the experiment

The overall structure of the experiment for all participants was as follows:

- Day 1—an auditory generalization pre-test
- Days 2 and 3—four sessions of training
- Day 4—a vocabulary test and an auditory identification test. (During the auditory identification test, Event Related Potentials (ERPs) were measured, but these results are not reported in the present paper).
- Day 5—an auditory generalization post-test

For Days 1, 4, and 5, all participants took the identical tests. For Days 2 and 3, each participant went through only one of the four types of training. At least 1 day and no more than 3 days separated any 2 Days of the experiment. For example, a participant had to schedule the first day of training (Day 2) at least 1 day but no more than 3 days after the auditory generalization pre-test (Day 1).

### Training materials

Training stimuli were ten pairs of Japanese words that contrasted in length of vowels [e eː o oː u uː] (Table 1). The materials included the contrast in the first syllable of the five word pairs and in the second syllable in other five word pairs. To increase variability of stimuli, these words were spoken (in isolation) twice, each with slow and fast speaking rates by two female native speakers of Japanese. The following instructions of the slow and fast speaking rates were given to the speakers: “a slow rate is slower than one's normal rate, clearly enunciating,” and “a fast speaking rate is faster than one's normal rate, but still comfortable and accurate.” To ensure naturalness, the actual rate of speech was determined by each speaker. A total of 80 audio files (10 word pairs × 2 lengths × 2 repetitions × 2 speakers) were used in the auditory portion of training (Step A in the training procedure section).

**Table 1:** **Training stimuli**.

**Contrasts in the first syllables**			**Contrasts in the second syllables**		
**Word**	**Length**	**Meaning**	**Word**	**Length**	**Meaning**
seki	SS	Seat	ɕaɾe	SS	Joke
seːki	LS	Century	ɕaɾeː	SL	Honorarium
kedo	SS	But	goke	SS	Widow
keːdo	LS	Slight degree	gokeː	SL	Word form
toɕo	SS	Book	joko	SS	Side
toːɕo	LS	At the beginning	jokoː	SL	Rehearsal
koɟʑi	SS	Orphan	iso	SS	Seashore
koːɟʑi	LS	Construction	isoː	SL	Transport
kuɾo	SS	Black	ɟʑiɕu	SS	To turn yourself in
kuːɾo	LS	Air path	ɟʑiɕuː	SL	Self-study

Words are written in an International Phonetic Alphabet phonetic transcription. Length SS refers to words with two short vowels; LS, words with a long vowel and a short vowel; SL, words with a short vowel and a long vowel.

In addition, 40 video clips (10 word pairs × 2 lengths × 2 speakers) were created by the same two speakers above to provide the visual dimension of our multimodal training (Steps D, F). For each video clip presenting short vowel words (e.g., [seki] or [joko]), the speaker spoke words and made the hand gesture of two small downward chopping movements. For words with long vowels (e.g., [seːki] or [jokoː]), two types of clips were made, one for the syllable condition and the other for the mora condition. For the syllable condition, the speaker's hand made one horizontal sweep for a long vowel (as in Roberge et al., 1996), followed or preceded by a small downward chopping movement for a short vowel, as they spoke a long-short or short-long word. For the mora condition, in contrast, the speaker's hand made two small downward chopping movements for a long vowel. Thus, they made three short vertical hand movements in long-vowel words (e.g., [seːki] or [jokoː]), and this hand gesture corresponds with the number of moras in those words. Note that gestures for short vowels are identical for the two conditions, with long vowels being the only part where the conditions diverge. Refer to Figure 1.

The two native Japanese speakers made 40 total video clips in which the 20 training words (Table 1) were spoken and gestured at slow and fast rates. The speaking rate was determined in the same way as when their audio recordings were made. The speakers used their right hand to gesture, and the videos were digitally flipped so that it appeared to be the left hand in order for participants to mirror the gestures they see with their own right hand.

The video clips showed the speaker's face speaking the word and the upper half of the body so that viewers could see hand movements and the face at the same time. In Hirata and Kelly (2010), visual information conveyed through lip movements played a significant role in auditory learning of Japanese vowel length contrasts, and although we did not isolate the lips as a variable in the present study, we wanted to explore the additive role of hand gesture by having both the mouth and hand visible in all conditions.

After auditory and video stimuli were created separately, the audio in the original video was deleted, and new audio clips (spoken without any hand gestures) were dubbed onto the video stimuli. It is known that the acoustic properties of speech are affected by co-speech hand gesture (Krahmer and Swerts, 2007), so it is possible that making the syllable and mora gestures would also alter their speech in subtle ways. By having identical auditory information across the four conditions, we assured that any differences in training would be attributed to gesture and not actual differences in the acoustic speech signal.

### Training procedure

Four sessions of training were conducted in Days 2 and 3 (see the overall structure section). At least 1 day and no more than 3 days separated Days 2 and 3 (consistent with the procedure in Hirata and Kelly, 2010). In each training session, participants went through 80 trials, i.e., the 20 words spoken by the two speakers repeated twice in a randomized order. Participants were exposed to only slow rate stimuli in Session 1, only fast rate stimuli in session 2, and both slow and fast rate stimuli in a randomized order in sessions 3 and 4.

**Figure 2:**
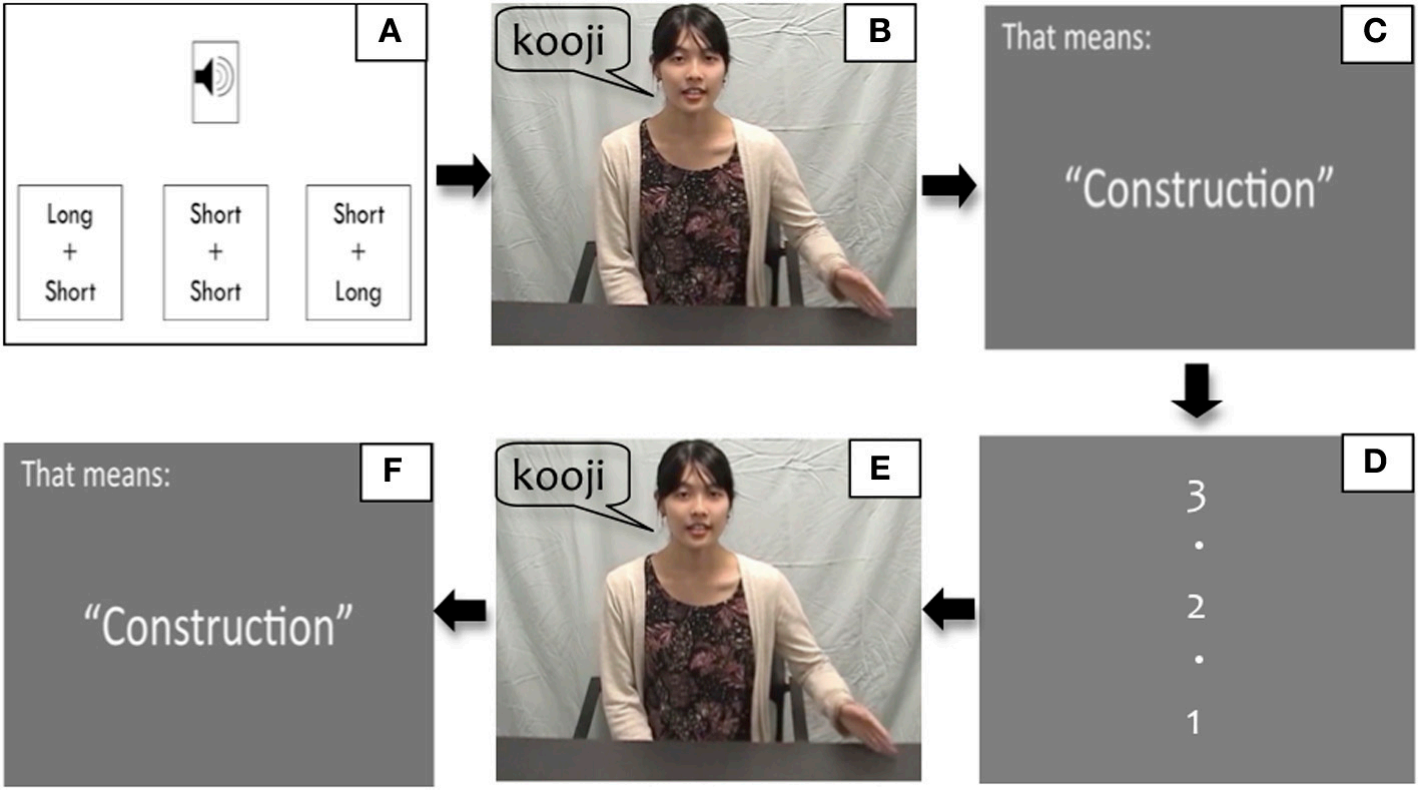
**Training steps. (A)** Listen to the target word audio, e.g., [koːji], and click one of the three alternatives, e.g., “Long + Short”; **(B)** Watch the instructor speaking the target word, e.g., [koːji], and showing syllable or mora gestures along with speech; **(C)** See the translation of the target word, e.g., “Construction”; **(D)** See the count down “3, 2, 1”; **(E)** Watch the same video as **(B)** and either observe or produce the respective gesture along with the video; **(F)** See the translation again.

The following steps were involved for each of the 80 trials (Figure 2):

Step A: Press the space bar to listen to the audio file of the word (produced by one of the two Japanese speakers), and choose one of the three alternatives that corresponded with what they heard: “short-short” (as in [koji] or [joko]), “long-short” (as in [koːji]), and “short-long” (as in [jokoː]) on the computer screen.
Step B: Watch a video clip in which the speaker said the word along with the accompanying hand gestures (which is indirect feedback regarding the correct answer to participants' response in Step A). Participants in Syllable and Mora conditions saw the syllable and mora gestures, respectively.
Step C: See an English translation of the word written on the screen, e.g., “That means ‘construction’.”
Step D: See a count down “3,” “2,” and “1” on the screen for 3 s.
Step E: Watch the same videos as in Step B. The participants in Syllable-Observe and Mora-Observe groups quietly observed the respective videos, and those in Syllable-Produce and Mora-Produce groups mimicked the respective gestures in the videos.
Step F: See the translation of the word again as in Step C.

The participants were instructed to be silent the whole time. Step A (to play the audio and to choose one of the three alternatives) was self-paced, but the other steps were automated by a computer program.

During training, the experimenters monitored the participants through a live video camera to assure that they adhered to their expected tasks in the four conditions. To motivate participants, they were told at the beginning of the first training session that the person who improved most in the test scores would receive a prize.

### Vocabulary test (on day 4)

The vocabulary test consisted of 30 words, including the 20 trained words and 10 distractor words. The distractors contained a phonetic composition of consonants and vowels that was identical to the trained words except for the length of the vowels. For example, [seki] “seat” and [seːki] “century” were trained words, and the distractor was [sekiː] (which is a nonsense word because of the length of the two vowels are switched). Materials were made up of (1) the identical audio files used in training, spoken at the slow rate by one of the speakers, and (2) the additional audio files of the distractor words, which were also spoken at the same slow rate by the same speaker. These 30 individual words were organized in a set randomized order, and each word was presented three times with a self-paced pause following each triplet.

The format of the vocabulary test was a free recall task, in which participants were asked to write down the meaning of words in English on a piece of paper, and to write down an “X” if they heard words that sounded similar to the trained words but that had different vowel length (i.e., distractor words). The test was self-paced, but participants were told not to go back to previous answers once they moved on to the later trials. The test took about 15 min for each participant to complete.

### Auditory identification test (on day 4)

Participants' auditory abilities were measured in two tests: an *auditory identification test* (on Day 4) and an *auditory generalization test* (on Days 1 and 5). The purpose of the auditory identification test was to measure participants' ability to immediately recognize the set of words that they had learned in training and to differentiate them from ones that sounded similar but were different from the trained words in terms of length of the vowels. An example of an untrained word would be [sekiː] for the word pair [seki] and [seːki], which was the same as the distractor words in the vocabulary test. The auditory identification test also included untrained words in which both syllables had long vowels, e.g., [seːkiː]. Thus, there was a total of 40 words used in this test, consisting of 20 trained and 20 untrained words.

These 40 words were each presented five times in a randomized order through a speaker. An automated program was created so that the inter-stimulus intervals were at random intervals between 2 and 3 s. The task for participants was a speeded 2-alternative forced identification: participants were asked to press one button as quickly as possible for words they had learned during training, and to press another button for new words that were not trained. This “old-new” format was chosen so that it was compatible with the method of measuring Event Related Potential (ERP) responses at the same time in order to examine how the brain responded to the trained vs. untrained words. Results from the ERP measure, however, will be reported in a separate paper.

### Auditory generalization tests (on days 1 and 5)

Auditory generalization tests consisted of a pre-test that was conducted before training on Day 1 and a post-test that was conducted after training on Day 5. The purpose of the auditory generalization tests was to measure changes in participants' generalized auditory ability to identify vowel length of *novel* words, rather than to accurately recognize the trained stimuli. Therefore, the words in the generalization tests were all different from those used in training, and each was presented in various carrier sentences produced by a novel female speaker of Japanese who was different from the speakers in the training sessions. The pre- and post-test each contained a total of 120 stimuli. There were 10 target disyllable pairs. For five of the word pairs, the vowel length contrasts were in the first syllable, e.g., [eki] “station” (short + short) vs. [eːki] “energetic spirit” (long + short), and for the other five word pairs, the contrasts were in the second syllable, e.g., [mizo] “ditch” (short + short) vs. [mizoː] “unprecedented” (short + long). These 20 words were the same for both of the pre-test and the post-test, but were spoken in different carrier sentences, e.g., *sore wa* ___ *da to omou* “I think that is ___.” Two carrier sentences used for the pre-test were different from those used for the post-test. Each of these materials was spoken at slow and fast speaking rates.

In order to eliminate any response bias, we needed to match the number of the following three types of words: “short + short,” e.g., [eki] and [mizo], “long + short,” e.g., [eːki], and “short + long,” e.g., [mizoː]. Of the 120 stimuli in each pre- or post-test, there were 40 “short + short” words (10 words × 2 rates × 2 sentences × 1 repetition), 40 “long + short” words (5 words × 2 rates × 2 sentences × 2 repetitions), and 40 “short + long” words (5 words × 2 rates × 2 sentences × 2 repetitions). By doing this, each item had an equal 33.33% chance of appearing. Half of participants in each of the four conditions heard carrier sentences 1 and 2 at the pre-test, and carrier sentences 3 and 4 at the post-test, and this order was switched for the other half of participants.

For each test, the stimuli described above were randomly presented across word pairs, carrier sentences, and speaking rates. Within each trial, a carrier sentence (e.g., “sore wa ___ da to omou”) was written on the computer screen. The participants' task was to listen to varying words inserted in the underlined location and to choose one of three alternatives, i.e., “short + short,” “long + short,” and “short + long,” that matched the vowel length pattern of those varying words. The trials were divided into six blocks for each test, and participants took a short break between blocks. Participants received no feedback on their performance at any time. The task was self-paced, and each test took about 20–30 min to complete. Participants took the auditory post-test within 1–3 days after their final training session.

## Results

### Vocabulary scores

Although participants learned the vocabulary words in all four conditions (chance performance is 5%), a one-way factorial ANOVA revealed that there were no significant differences across the instruction groups, *F*_(3, 84)_ = 0.436, ns. Refer to Table 2.

**Table 2:** **Vocabulary scores across the four instruction conditions**.

	**Syllable observe**	**Syllable produce**	**Mora observe**	**Mora produce**
Vocabulary score	0.73 (0.28)	0.67 (0.31)	0.77 (0.23)	0.72 (0.33)

The numbers are proportion correctly recalled, followed by the SDs (in parentheses).

### Auditory identification scores and reaction times

**Figure 3:**
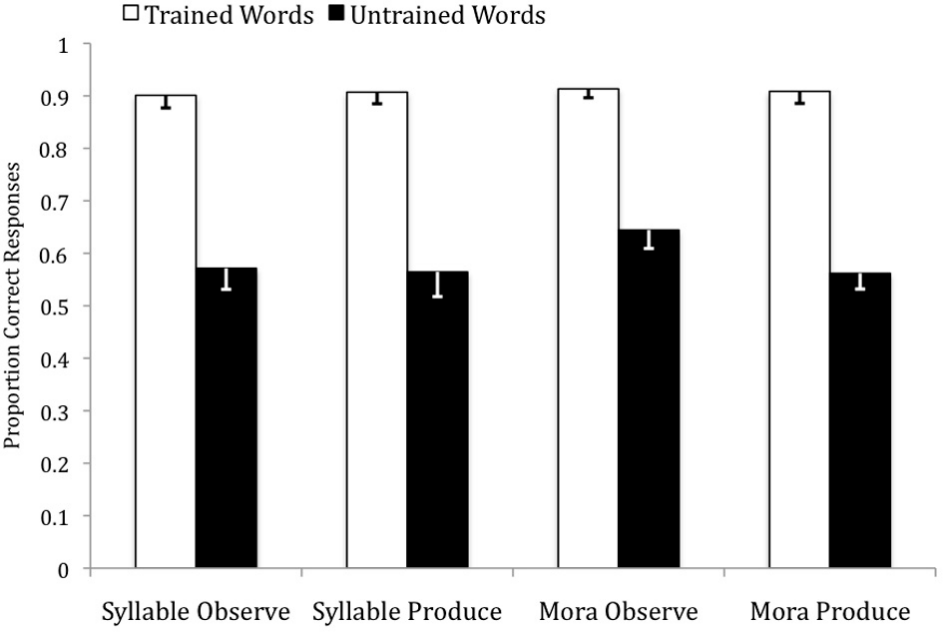
**Accuracy rates for trained and untrained words across the four instruction conditions in the auditory identification test**.

The accuracy rates and reaction times (RTs) were subjected to two separate 2 (trained, untrained) by 4 (SO, SP, MO, MP) mixed ANOVAs^2^. For the accuracy rates, there was no main effect of instruction condition, *F*_(3, 79)_ = 0.24, ns, but there was a significant main effect of word type, *F*_(1, 79)_ = 281.27, *p* < 0.001, η^2^_*p*_ = 0.78, with trained items (*M* = 0.91, *SD* = 0.10) producing higher accuracy rates than untrained items (*M* = 0.59, *SD* = 0.18) across all instruction conditions. Within each instruction condition, these differences were all significant at the *p* < 0.001 level. There was no significant word type by instruction condition interaction, *F*_(3, 79)_ = 0.84, ns. Refer to Figure 3.

**Figure 4:**
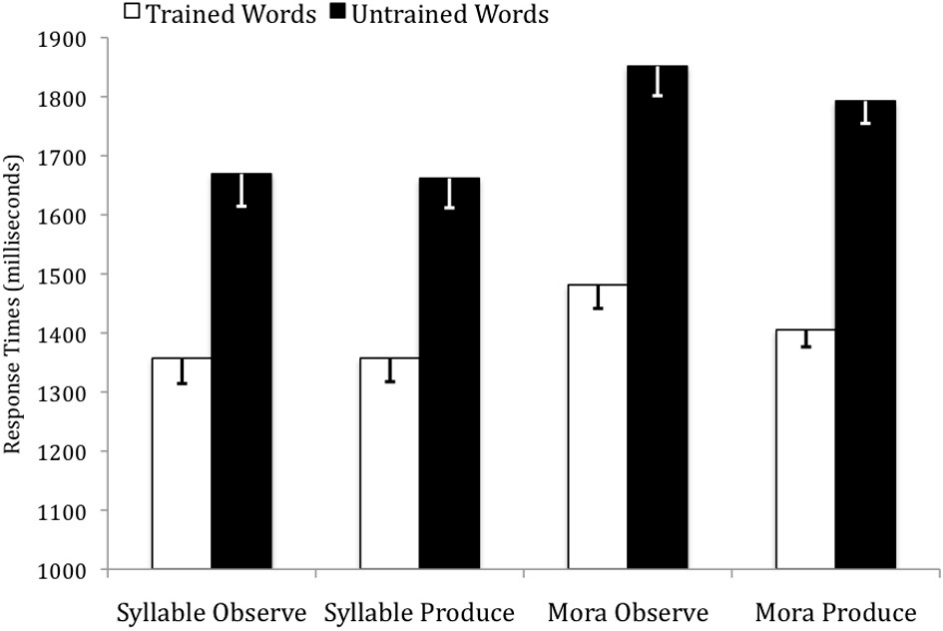
**Response times for trained and untrained words across the four instruction conditions in the auditory identification test**.

For the RTs, there was a main effect of instruction condition, *F*_(3, 79)_ = 3.32, *p* = 0.024, η^2^_*p*_ = 0.11. Visual inspection of the data suggested that the two Mora conditions (*M* = 1633 ms, *SD* = 169 ms) produced slower RTs than the two Syllable conditions (*M* = 1512 ms, *SD* = 204 ms), and this difference was significant *F*_(1, 81)_ = 8.68, *p* = 0.004, η^2^_*p*_ = 0.10. In addition, there was a significant main effect of word type, *F*_(1, 79)_ = 507.06, *p* < 0.001, η^2^_*p*_ = 0.86, with trained items (*M* = 1401 ms, *SD* = 180 ms) producing faster RTs than untrained items (*M* = 1745 ms, *SD* = 233 ms) across all instruction conditions. Within each instruction condition, these differences were all significant at the *p* < 0.001 level. There was no significant word type by instruction condition interaction, *F*_(3, 79)_ = 1.83, ns. Refer to Figure 4.

### Auditory generalization scores

The scores on the auditory generalization test were subjected to two a 2 (pre-test, post-test) by 4 (SO, SP, MO, MP) mixed ANOVA. There was a main effect of test time, *F*_(1, 84)_ = 66.34, *p* < 0.001, η^2^_*p*_ = 0.44, with participants in all instruction groups improving from pre- (*M* = 0.70, *SD* = 0.15) to post-test (*M* = 0.80, *SD* = 0.14). Within each instruction condition, these differences were all significant at the *p* < 0.001 level. However, there was no significant main effect of instruction, *F*_(3, 84)_ = 0.02, ns, or interaction of test time and instruction condition, *F*_(3, 84)_ = 0.04, ns. Refer to Table 3.

**Table 3:** **Pre- and post-test scores from the generalization auditory test across the four instruction conditions**.

**Condition**	**Pre-test**	**Post-test**
Syllable observe	0.70 (0.14)	0.79 (0.13)
Syllable produce	0.70 (0.16)	0.80 (0.13)
Mora observe	0.71 (0.18)	0.80 (0.15)
Mora produce	0.70 (0.14)	0.79 (0.16)

The numbers are proportion correct, followed by the SDs (in parentheses).

### Correlations among vocabulary and auditory scores

In order to investigate whether participants actually applied their auditory learning to performing the vocabulary task, we ran correlations among the vocabulary scores, RTs and accuracy scores for the auditory identification test, and the pre- and post-test auditory generalization scores. In general, vocabulary scores were positively correlated with almost all measures of auditory processing. In particular, note that performance in the auditory identification test accounts for much variance (over 40% for accurately identifying trained items) in the vocabulary performance. Also note that the auditory generalization scores, to a lesser extent, accounts for a sizeable portion of that variance as well^3^. Although both the pre- and post-test both account for significant variance in vocabulary performance (~25 and 20%, respectively), the post-test accounts for significantly more variance when comparing betas in a multiple-regression analysis, pre-test beta: 5.42, *t* = 1.01, ns; post-test beta: 16.39, *t* = 2.82, *p* = 0.006. Refer to Table 4.

**Table 4:** **Correlation coefficients among the vocabulary scores, response times (RT trained, RT untrained) and accuracy scores for the auditory identification scores (Accuracy trained, Accuracy untrained) and the pre- and post-test auditory generalization scores (Pre-test auditory, Post-test auditory)**.

	**Pre-test auditory**	**Post-test auditory**	**Accuracy trained**	**Accuracy untrained**	**RT trained**	**RT untrained**
Vocabulary score	0.454^**^	0.515^**^	0.640^**^	0.395^**^	0.021	0.270^*^
Pre-test auditory	–	0.764^**^	0.422^**^	0.441^**^	0.101	0.196
Post-test auditory	–	–	0.451^**^	0.458^**^	−0.013	0.178
Accuracy trained	–	–	–	0.263^*^	0.006	0.299^**^
Accuracy untrained	–	–	–	–	−0.194	−0.218^*^
RT trained	–	–	–	–	–	0.803^**^

* p < 0.05;

** p < 0.01 (two-tailed).

## Discussion

### Limited role of hand gestures

We did not find support for our first two predictions in any of the three sets of dependent measures. In none of our measures—vocabulary, auditory identification, and auditory generalization—did the mora and produce conditions out-perform the syllable and observe conditions, respectively. This null finding is interesting in light of the fact that our phoneme and vocabulary training was, overall, highly effective. Participants learned vocabulary at a high rate (roughly 70% correct recall), far exceeding chance performance, and all groups improved from pre- to post-test in their ability to distinguish novel phoneme contrasts in the auditory generalization task [similar to previous work, see Hirata et al. (2007)]. Finally, the positive correlations between the two auditory tasks and the vocabulary task suggest that participants were using their newly acquired phoneme discrimination abilities to remember word meanings, which requires, at a fundamental level, the ability to discriminate long and short vowels. These significant effects also rule out the possibility that we simply did not have enough power to uncover differences across our training conditions. To the contrary, we had moderate-to-large effect sizes in the comparison between pre- and post-tests for the auditory generalization task and very large effect sizes for the RTs and error rates in correctly identifying trained and untrained words in the auditory identification task.

Although one needs to be careful when interpreting null results, the present findings seem to tell a clear story: observing and producing different types of hand gestures *does not* help with learning Japanese long and short vowel distinctions and word meanings comprised of those distinctions. This story is consistent with similar result from a previous study using a comparable training paradigm (Hirata and Kelly, 2010). As described in the introduction, participants in that study were trained to make short and long vowel distinctions in Japanese by observing the same sorts of “syllable gestures” used in this study. The “Observe Syllable” condition in that study (called the Audio + Mouth + Hands condition) produced an improvement of 5% points, which was statistically indistinguishable from a baseline condition of Auditory Only training, which produced a 7% improvement. It is difficult to compare across studies, but it is interesting that the average improvement for all training conditions in the present study (~9%) was very similar to the Auditory Only improvement in the Hirata and Kelly study.

At first blush, the findings from these two studies are surprising in light of the well-established research—much of it discussed in this special issue—on the benefits of multimodal processing and learning (Calvert et al., 2004). For example, focusing on mouth movements and speech perception, neuroimaging research has shown that visual information from the lips interacts with speech perception at early stages (Klucharev et al., 2003; Besle et al., 2004) and enhances processing in primary visual and auditory cortices compared to visual and auditory input alone (Calvert et al., 1999). With specific regard to L2 learning, research has shown that language learners benefit from instruction that includes speech and visual mouth movements compared to just speech alone (Hardison, 2005; Wang et al., 2008; Hirata and Kelly, 2010).

More recently, researchers have expanded their focus on multimodal communication to include not just the face, but the whole body as well. Indeed, there is growing research on the role of observing and producing hand gestures in language processing and learning (Kelly et al., 2008; Goldin-Meadow, 2014). For example, beat gestures (quick flicks of the hand emphasizing certain words) can change how listeners perceive words (Krahmer and Swerts, 2007; Biau and Soto-Faraco, 2013), and this change in perception is caused by increased activity in auditory brain regions (Hubbard et al., 2008). Moreover, in the context of L2 learning, observing (Kelly et al., 2009) and producing (Macedonia et al., 2011) iconic hand gestures helps to learn and remember new vocabulary in a foreign language.

Given this work on the benefits of multimodal input in language processing and learning, why would observing and producing different types of gesture not help in the present study? We hypothesize that gestures may not be “built for” work at the level in which we applied them. Our gestures were designed to be a visual metaphor of a subtle auditory distinction *within a syllable* at the segmental level. This “within syllable” auditory distinction may be better captured by lip movements, which have a more natural and direct correspondence to the speech they produce. In contrast, gestures may not be easily mapped onto to such small units within a word. Things change when one moves beyond the word level to the sentence level. Indeed, gestures work very well to emphasize the semantically most relevant words within the context of a sentence (Krahmer and Swerts, 2007).

Of course, another explanation for our results is that that gestures do function to make phonemic distinctions within syllables, but just not the phonemic *length* distinctions studied in the present experiment. Recall that the reason English speakers struggle with distinguishing long and short vowels in Japanese is that in English, vowel length is not phonemic—that is, the length of a vowel alone does not change the meaning of a word (Vance, 1987). Indeed, Hirata (2004b) has shown that, for novice English-speaking learners of Japanese, these length distinctions are very hard to learn. Considering this, the auditory contrast may simply be just too foreign and unusual to the novice ear of an English speaker. In contrast, it would be interesting to explore whether gestures play a significant role in learning other types of L2 phonemic contrasts. For example, tones are phonemic in Mandarin, and it would be interesting to examine whether hand gestures that exploit rising and falling space imitating the tonal contours would help L2 learners to hear the tonal distinction. Another example might be the distinction of different vowel types such as English vowels in *collar* vs. *color*, and it would be interesting to examine whether L2 learners of English would benefit from training with fingers wide open vs. closer together to represent the relative size of the mouth opening.

### Mora vs. syllable gestures

One unexpected finding was that for the auditory identification task, people were significantly slower (across the Observe and Produce conditions) to correctly identify trained and untrained words in the two Mora conditions compared to the two Syllable conditions. This finding is notable for a few reasons. First, it demonstrates that there was indeed enough power to uncover significant effects of our training conditions for our different dependent measures. Second, it suggests that, if anything, the mora gestures made the task of identifying trained and untrained words more difficult than syllable gestures—indeed, participants in the two Mora instruction conditions were over 100 ms slower in correctly identifying words than the Syllable condition. This finding provides validation that Mora gestures may indeed be “non-intuitive” to English-speakers, but contrary to this “mismatching” information helping learners, processing them appears to slow learners down. In this way, the Mora gestures act less like the “mismatching” gestures that have been shown to help with learning (Goldin-Meadow, 2014) and more like the “incongruent” gestures that have been shown to slow processing of speech information (Kelly et al., 2010) and disrupt memory for newly learned L2 vocabulary (Kelly et al., 2009).

It would be interesting to explore how more advanced learners of Japanese would react to mora and syllable gestures. Given their more extensive experience with Japanese and better grasp of phonemic distinction between long and short vowels, one might predict that they may have an easier time processing words learned with mora gestures. This raises the interesting possibility that L2 learners may benefit from different types of multimodal input at different stages of learning.

### Implications and conclusion

These findings have important implications for L2 language instruction. We already know from previous research that multimodal input can be very useful when teaching L2 learners novel speech sounds (Hardison, 2003, 2005; Wang et al., 2008; Hirata and Kelly, 2010). These studies have all shown that presenting congruent lip movements with auditory phoneme instruction helps people learn novel phoneme contrasts above and beyond auditory input alone. However, there is evidence that layering *too much* multimodal information onto novel speech sounds may over-load the system and actually produce decrements in perception and learning (Hirata and Kelly, 2010; Kelly and Lee, 2012). For example, Hirata and Kelly (2010) showed that whereas seeing lip movements with speech helped English learners to distinguish Japanese long and short vowels better than speech alone, adding hand gestures to lip and audio training actually removed the positive effects of the mouth.

The present findings add an interesting layer to these studies. When learners have difficulty mapping the meaning of gestures onto novel speech sounds (as with metaphoric gestures conveying information about length of phonemes), it may be wise to eliminate this form of multimodal input from the instruction process, and instead, provide visual input only from the lips and mouth. In contrast, when learners have better mastery with L2 speech sounds, it may be helpful to add gestural input, especially when teaching vocabulary (Quinn-Allen, 1995) and grammar (Holle et al., 2012). So it appears that more multimodal input is not always better in L2 instruction (Hirata and Kelly, 2010; Kelly and Lee, 2012). It will be important to continue this sort of systematic research to carefully demarcate not only what components of second language learning benefit from multimodal input, but also what types of multimodal input optimally enhance those specific components.

Finally, these results are useful in fleshing out claims that gesture and speech constitute an *integrated system* (McNeill, 1992, 2005; Kendon, 2004). For example, McNeill argues that gesture and speech are deeply intertwined and both stem from the same “Growth Point,” which he identifies as the conceptual origin of all utterances. When someone gestures, that gesture manifests the most relevant (or “newsworthy,” to use McNeill's term) imagistic information contained in that starting point, whereas the speech handles the more traditional functions of language, i.e., the linear, segmentable, and conventional components. Thus, gestures visually highlight information that is conceptually essential to the meaning of an utterance. There is support for this relationship of gesture to speech in the literature on language comprehension (Kelly et al., 2004; Willems et al., 2007; Hostetter, 2011), but the present study suggests that this integrated system may not operate at lower levels of language processing. Perhaps because gestures are so well suited for highlighting semantically relevant information at the utterance level, it is unnatural for them to draw attention to lower level phonemic information *at the segmental timing level*. It will be important for future research on gesture comprehension to more carefully delineate what aspects of gestures form a tightly integrated system with speech—and what aspects do not.

### Conflict of interest statement

The authors declare that the research was conducted in the absence of any commercial or financial relationships that could be construed as a potential conflict of interest.

## Footnotes

^1^These gestures were described as “beats” in the Kelly and Hirata paper because of their rhythmic properties. However, these gestures could also be described as “metaphoric” because they cross-modally mapped lengths of visual movements onto lengths of auditory distinctions. Although the gestures used in the present study could also be categorized as both of these types of gesture, we will refer to them as metaphorics.

^2^Five participants were excluded from this analysis because of technical difficulties with the program collecting the error rates and RTs.

^3^When pitted against each other in a multiple regression analysis, accuracy in identifying trained items accounts for significantly more variance than pre- and post-test generalization scores.
